# *N*′-[(*E*)-2-Hy­droxy-5-meth­oxy­benzyl­idene]pyridine-4-carbohydrazide

**DOI:** 10.1107/S1600536810043382

**Published:** 2010-10-30

**Authors:** Hadi Kargar, Reza Kia, Mehmet Akkurt, Orhan Büyükgüngör

**Affiliations:** a Department of Chemistry, School of Science, Payame Noor University, Ardakan, Yazd, Iran; b Department of Chemistry, Science and Research Branch, Islamic Azad University, Tehran, Iran; c Department of Physics, Faculty of Arts and Sciences, Erciyes University, 38039 Kayseri, Turkey; d Department of Physics, Faculty of Arts and Sciences, Ondokuz Mayıs University, 55139 Samsun, Turkey

## Abstract

In the title compound, C_14_H_13_N_3_O_3_, the dihedral angle between the pyridine and benzene rings is 15.17 (18)°. The torsion angle of the –C=N—N—C– system between two aromatic rings is −167.1 (3)°. Intra­molecular O—H⋯N hydrogen bonding generates *S*(6) rings. In the crystal structure, neighbouring mol­ecules are linked together along the *c* axis by weak inter­molecular C—H⋯O and N—H⋯O hydrogen bonds, generating *R*_1_^2^(6) ring motifs.

## Related literature

For the tuberculostatic activity of isoniazid (isonicotinylhydrazine) derivatives, see: Janin (2007 ▶); Maccari *et al.* (2005 ▶). For the synthesis of the isoniazid derivative, see: Lourenco *et al.* (2008 ▶). For hydrogen-bond motifs, see: Bernstein *et al.* (1995 ▶).
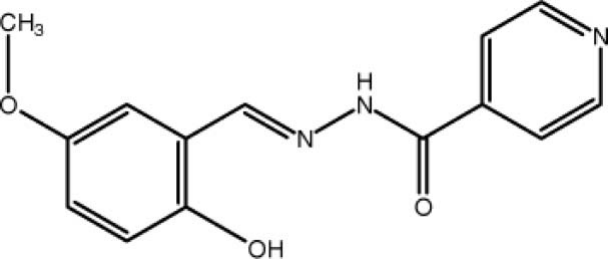


## Experimental

### 

#### Crystal data

C_14_H_13_N_3_O_3_
*M* *_r_* = 271.27
Monoclinic, 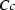

*a* = 6.1114 (6) Å
*b* = 29.489 (3) Å
*c* = 7.4820 (7) Å
β = 96.696 (8)°
*V* = 1339.2 (2) Å^3^
*Z* = 4
Mo *K*α radiation
μ = 0.10 mm^−1^
*T* = 296 K
0.67 × 0.34 × 0.12 mm

#### Data collection

Stoe IPDS 2 diffractometer
Absorption correction: integration (*X-RED32*; Stoe & Cie, 2002 ▶) *T*_min_ = 0.962, *T*_max_ = 0.988
4353 measured reflections
1542 independent reflections
1166 reflections with *I* > 2σ(*I*)
*R*_int_ = 0.038

#### Refinement

*R*[*F* ^2^ > 2σ(*F* ^2^)] = 0.046
*wR*(*F* ^2^) = 0.114
*S* = 1.11
1542 reflections
191 parameters
2 restraints
H atoms treated by a mixture of independent and constrained refinement
Δρ_max_ = 0.13 e Å^−3^
Δρ_min_ = −0.16 e Å^−3^

### 

Data collection: *X-AREA* (Stoe & Cie, 2002 ▶); cell refinement: *X-AREA*; data reduction: *X-RED32* (Stoe & Cie, 2002 ▶); program(s) used to solve structure: *SIR97* (Altomare *et al.*, 1999 ▶); program(s) used to refine structure: *SHELXL97* (Sheldrick, 2008 ▶); molecular graphics: *ORTEP-3* (Farrugia, 1997 ▶); software used to prepare material for publication: *WinGX* (Farrugia, 1999 ▶).

## Supplementary Material

Crystal structure: contains datablocks global, I. DOI: 10.1107/S1600536810043382/sj5051sup1.cif

Structure factors: contains datablocks I. DOI: 10.1107/S1600536810043382/sj5051Isup2.hkl

Additional supplementary materials:  crystallographic information; 3D view; checkCIF report

## Figures and Tables

**Table 1:** Hydrogen-bond geometry (Å, °)

*D*—H⋯*A*	*D*—H	H⋯*A*	*D*⋯*A*	*D*—H⋯*A*
O1—H1⋯N1	0.96 (4)	1.80 (4)	2.628 (4)	142 (4)
N2—H2⋯O3^i^	0.88 (4)	2.03 (4)	2.872 (4)	161 (4)
C8—H8⋯O3^i^	0.93	2.43	3.188 (4)	139

Symmetry code: (i) 
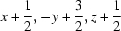
.

**Fig. 1.**
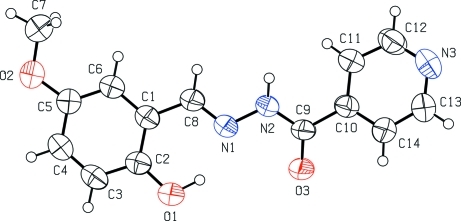
The title molecule, with the atom numbering scheme. Displacement ellipsoids for non-H atoms are drawn at the 50% probability level.

**Fig. 2.**
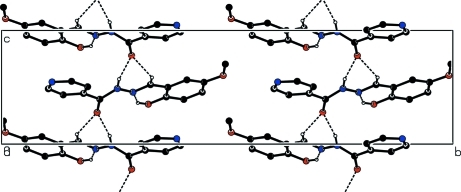
The packing and hydrogen bonding interactions of (I), down a axis, showing R12(6) ring motifs. All H atoms not involved in hydrogen bonding are omitted for clarity.

## supplementary crystallographic information

### Comment

In the search for new biologically active compounds, isoniazid (isonicotinylhydrazine) derivatives have been found to possess potential tuberculostatic activity (Janin, 2007; Maccari *et al.*, 2005). Here, we present the crystal structure of the title compound, (I).

In the title compound (I), (Fig. 1), the dihedral angle between the pyridine (C10–C14) and benzene (C1–C6) rings is 15.17 (18)°. The C8–N1–N2–C9 torsion angle is -167.1 (3) °.

Intramolecular O1—H1···N1 hydrogen bonding generates *S*(6) rings (Bernstein *et al.*, 1995) (Table 1, Fig. 2). In the crystal structure, neighbouring molecules are linked together by weak intermolecular C—H···O and N—H···O hydrogen bonds (Table 1, Fig. 2), generating *R*_1_^2^(6) ring motifs (Bernstein *et al.*, 1995), and linking the molecules along the *c* axis.

### Experimental

The isoniazid derivative was prepared following procedure reported by Lourenco *et al.*, (2008). 5-methoxysalicylaldehyde (1.0 mmol) was added to isoniazid (1.0 mmol) in ethanol. After stirring for 3 h at reflux condition, the resulting mixture was concentrated at room temperature. The residue was purified by washing with cold ethanol and diethyl ether to give the pure derivative. Colourless single crystals suitable for X-ray analysis were obtained by re-crystallization from methanol.

### Refinement

In the absence of significant anomalous dispersion effects, Freidel pairs were merged. The calculation of the Flack (1983) parameter was suppressed by the MERG 4 command in *SHELXL*97 (Sheldrick, 2008), as the lack of anomalous scatterers did not allow the determination of the absolute configuration from the X-ray measurements. The H atoms of the O—H and N—H groups were found from a difference Fourier map and refined freely [O1—H1 = 0.96 (4) and N2—H2 = 0.88 (4) Å]. The remaining H atoms were placed in idealized positions and allowed to ride on their parent atoms, with C—H = 0.93 and 0.96 Å for aromatic and methyl H, respectively, and *U*_iso_(H) = 1.5*U*_eq_(C) for methyl H and *U*_iso_(H) = 1.2*U*_eq_(C) for aromatic H.

### Crystal data

**Table:**

C_14_H_13_N_3_O_3_	*F*(000) = 568
*M**_r_* = 271.27	*D*_x_ = 1.345 Mg m^−^^3^
Monoclinic, *C**c*	Mo *K*α radiation, λ = 0.71073 Å
Hall symbol: C -2yc	Cell parameters from 7613 reflections
*a* = 6.1114 (6) Å	θ = 2.8–28.0°
*b* = 29.489 (3) Å	µ = 0.10 mm^−^^1^
*c* = 7.4820 (7) Å	*T* = 296 K
β = 96.696 (8)°	Prism, colourless
*V* = 1339.2 (2) Å^3^	0.67 × 0.34 × 0.12 mm
*Z* = 4	

### Data collection

**Table:**

Stoe IPDS 2 diffractometer	1542 independent reflections
Radiation source: sealed X-ray tube, 12 x 0.4 mm long-fine focus	1166 reflections with *I* > 2σ(*I*)
plane graphite	*R*_int_ = 0.038
Detector resolution: 6.67 pixels mm^-1^	θ_max_ = 27.5°, θ_min_ = 2.8°
ω scans	*h* = −7→7
Absorption correction: integration (*X-RED32*; Stoe & Cie, 2002)	*k* = −38→35
*T*_min_ = 0.962, *T*_max_ = 0.988	*l* = −9→8
4353 measured reflections	

### Refinement

**Table:**

Refinement on *F*^2^	Secondary atom site location: difference Fourier map
Least-squares matrix: full	Hydrogen site location: inferred from neighbouring sites
*R*[*F*^2^ > 2σ(*F*^2^)] = 0.046	H atoms treated by a mixture of independent and constrained refinement
*wR*(*F*^2^) = 0.114	*w* = 1/[σ^2^(*F*_o_^2^) + (0.0582*P*)^2^] where *P* = (*F*_o_^2^ + 2*F*_c_^2^)/3
*S* = 1.11	(Δ/σ)_max_ < 0.001
1542 reflections	Δρ_max_ = 0.13 e Å^−^^3^
191 parameters	Δρ_min_ = −0.16 e Å^−^^3^
2 restraints	Extinction correction: *SHELXL97* (Sheldrick, 2008), Fc^*^=kFc[1+0.001xFc^2^λ^3^/sin(2θ)]^-1/4^
Primary atom site location: structure-invariant direct methods	Extinction coefficient: 0.0090 (19)

### Special details

**Table:**

Geometry. Bond distances, angles *etc*. have been calculated using the rounded fractional coordinates. All su's are estimated from the variances of the (full) variance-covariance matrix. The cell e.s.d.'s are taken into account in the estimation of distances, angles and torsion angles
Refinement. Refinement on *F*^2^ for ALL reflections except those flagged by the user for potential systematic errors. Weighted *R*-factors *wR* and all goodnesses of fit *S* are based on *F*^2^, conventional *R*-factors *R* are based on *F*, with *F* set to zero for negative *F*^2^. The observed criterion of *F*^2^ > σ(*F*^2^) is used only for calculating -*R*-factor-obs *etc*. and is not relevant to the choice of reflections for refinement. *R*-factors based on *F*^2^ are statistically about twice as large as those based on *F*, and *R*-factors based on ALL data will be even larger.

### Fractional atomic coordinates and isotropic or equivalent isotropic displacement parameters (Å^2^)

**Table:**

	*x*	*y*	*z*	*U*_iso_*/*U*_eq_	
O1	−0.1045 (4)	0.82503 (10)	0.3515 (4)	0.0727 (9)	
O2	0.2101 (5)	0.99107 (9)	0.6041 (5)	0.0844 (10)	
O3	0.2501 (4)	0.71171 (8)	0.2791 (4)	0.0743 (9)	
N1	0.2870 (4)	0.79115 (10)	0.4570 (4)	0.0619 (9)	
N2	0.4398 (4)	0.75656 (9)	0.4882 (4)	0.0622 (9)	
N3	0.8707 (5)	0.61195 (13)	0.5468 (5)	0.0794 (11)	
C1	0.1994 (5)	0.86852 (12)	0.5002 (4)	0.0572 (10)	
C2	−0.0172 (5)	0.86497 (12)	0.4151 (5)	0.0599 (11)	
C3	−0.1453 (5)	0.90380 (14)	0.3930 (6)	0.0753 (15)	
C4	−0.0645 (6)	0.94459 (14)	0.4568 (6)	0.0766 (13)	
C5	0.1486 (5)	0.94855 (12)	0.5445 (5)	0.0646 (11)	
C6	0.2792 (5)	0.91072 (12)	0.5652 (5)	0.0611 (11)	
C7	0.4191 (7)	0.99583 (16)	0.7042 (7)	0.0830 (14)	
C8	0.3464 (5)	0.82985 (12)	0.5228 (5)	0.0591 (11)	
C9	0.4075 (4)	0.71774 (11)	0.3949 (4)	0.0574 (10)	
C10	0.5728 (5)	0.68156 (11)	0.4456 (4)	0.0564 (10)	
C11	0.7920 (5)	0.69069 (13)	0.5046 (5)	0.0672 (11)	
C12	0.9319 (6)	0.65507 (17)	0.5525 (6)	0.0778 (15)	
C13	0.6605 (6)	0.60362 (13)	0.4866 (6)	0.0739 (14)	
C14	0.5073 (5)	0.63664 (12)	0.4332 (5)	0.0655 (11)	
H1	0.007 (7)	0.8019 (15)	0.363 (6)	0.080 (12)*	
H2	0.552 (7)	0.7604 (14)	0.572 (6)	0.079 (11)*	
H3	−0.28770	0.90210	0.33400	0.0900*	
H4	−0.15340	0.97020	0.44150	0.0920*	
H6	0.42200	0.91300	0.62290	0.0730*	
H7A	0.53150	0.98860	0.62920	0.1250*	
H7B	0.43020	0.97560	0.80540	0.1250*	
H7C	0.43790	1.02650	0.74600	0.1250*	
H8	0.48570	0.83320	0.58600	0.0710*	
H11	0.84350	0.72040	0.51180	0.0810*	
H12	1.07860	0.66170	0.59120	0.0940*	
H13	0.61430	0.57360	0.48040	0.0880*	
H14	0.36330	0.62890	0.38980	0.0790*	

### Atomic displacement parameters (Å^2^)

**Table:**

	*U*^11^	*U*^22^	*U*^33^	*U*^12^	*U*^13^	*U*^23^
O1	0.0555 (12)	0.0673 (16)	0.0905 (19)	0.0003 (11)	−0.0119 (12)	−0.0055 (13)
O2	0.0782 (15)	0.0598 (16)	0.111 (2)	0.0057 (12)	−0.0066 (15)	−0.0122 (16)
O3	0.0669 (13)	0.0602 (14)	0.0864 (19)	0.0021 (11)	−0.0310 (12)	−0.0050 (13)
N1	0.0523 (12)	0.0570 (17)	0.0721 (19)	0.0056 (12)	−0.0115 (12)	0.0015 (14)
N2	0.0541 (12)	0.0544 (16)	0.0716 (18)	0.0064 (12)	−0.0195 (12)	−0.0042 (14)
N3	0.0687 (18)	0.079 (2)	0.090 (2)	0.0237 (16)	0.0069 (15)	0.0059 (19)
C1	0.0511 (15)	0.061 (2)	0.0581 (19)	0.0045 (13)	0.0008 (13)	0.0026 (15)
C2	0.0478 (14)	0.065 (2)	0.066 (2)	−0.0007 (14)	0.0023 (13)	0.0000 (17)
C3	0.0481 (17)	0.078 (3)	0.097 (3)	0.0108 (16)	−0.0031 (18)	0.003 (2)
C4	0.0616 (18)	0.069 (2)	0.096 (3)	0.0148 (17)	−0.0043 (18)	−0.002 (2)
C5	0.0606 (17)	0.058 (2)	0.074 (2)	0.0017 (15)	0.0028 (15)	−0.0040 (18)
C6	0.0495 (15)	0.062 (2)	0.070 (2)	0.0014 (13)	−0.0011 (14)	−0.0002 (16)
C7	0.081 (2)	0.072 (2)	0.093 (3)	−0.0038 (19)	−0.003 (2)	−0.011 (2)
C8	0.0519 (14)	0.059 (2)	0.063 (2)	0.0013 (13)	−0.0081 (13)	0.0011 (16)
C9	0.0458 (13)	0.0562 (19)	0.066 (2)	−0.0026 (12)	−0.0107 (13)	0.0011 (16)
C10	0.0504 (14)	0.0602 (19)	0.0565 (18)	0.0030 (13)	−0.0027 (13)	−0.0032 (16)
C11	0.0520 (15)	0.070 (2)	0.077 (2)	0.0048 (15)	−0.0028 (15)	−0.0028 (19)
C12	0.0548 (16)	0.090 (3)	0.087 (3)	0.0123 (18)	0.0011 (17)	−0.001 (2)
C13	0.074 (2)	0.061 (2)	0.086 (3)	0.0101 (17)	0.007 (2)	−0.0042 (19)
C14	0.0616 (17)	0.058 (2)	0.076 (2)	0.0017 (14)	0.0039 (16)	−0.0093 (18)

### Geometric parameters (Å, °)

**Table:**

O1—C2	1.356 (5)	C5—C6	1.370 (5)
O2—C5	1.369 (5)	C9—C10	1.488 (4)
O2—C7	1.410 (6)	C10—C11	1.387 (4)
O3—C9	1.230 (4)	C10—C14	1.384 (5)
O1—H1	0.96 (4)	C11—C12	1.375 (6)
N1—N2	1.384 (4)	C13—C14	1.377 (5)
N1—C8	1.279 (5)	C3—H3	0.9300
N2—C9	1.343 (4)	C4—H4	0.9300
N3—C13	1.334 (5)	C6—H6	0.9300
N3—C12	1.325 (6)	C7—H7A	0.9600
N2—H2	0.88 (4)	C7—H7B	0.9600
C1—C6	1.403 (5)	C7—H7C	0.9600
C1—C2	1.404 (4)	C8—H8	0.9300
C1—C8	1.449 (5)	C11—H11	0.9300
C2—C3	1.386 (5)	C12—H12	0.9300
C3—C4	1.365 (6)	C13—H13	0.9300
C4—C5	1.393 (5)	C14—H14	0.9300
			
O1···N1	2.628 (4)	C7···H6	2.5200
O1···C10^i^	3.347 (4)	C8···H1	2.42 (4)
O2···C7^ii^	3.409 (6)	C11···H2	2.61 (4)
O3···N2^i^	2.872 (4)	C12···H3^viii^	3.0600
O3···C12^iii^	3.418 (5)	C14···H12^iii^	3.0900
O3···N1	2.691 (4)	H1···N1	1.80 (4)
O3···C8^i^	3.188 (4)	H1···C8	2.42 (4)
O1···H12^iv^	2.6100	H2···C11	2.61 (4)
O2···H13^v^	2.6500	H2···H8	2.1900
O2···H7B^ii^	2.9100	H2···H11	2.2300
O3···H14	2.6400	H2···O3^vi^	2.03 (4)
O3···H2^i^	2.03 (4)	H3···N3^iv^	2.8500
O3···H8^i^	2.4300	H3···C12^iv^	3.0600
N1···O1	2.628 (4)	H4···H7A^iii^	2.5700
N1···O3	2.691 (4)	H6···C7	2.5200
N1···C11^i^	3.431 (5)	H6···H7A	2.3300
N2···O3^vi^	2.872 (4)	H6···H7B	2.2900
N1···H1	1.80 (4)	H6···H8	2.4100
N2···H11	2.6700	H7A···C6	2.7800
N3···H7C^vii^	2.9300	H7A···H4^xi^	2.5700
N3···H3^viii^	2.8500	H7A···H6	2.3300
C1···C14^ix^	3.576 (5)	H7B···C6	2.7100
C5···C7^ii^	3.590 (6)	H7B···H6	2.2900
C6···C13^ix^	3.344 (6)	H7B···O2^x^	2.9100
C7···O2^x^	3.409 (6)	H7C···C5^x^	3.0900
C7···C5^x^	3.590 (6)	H7C···N3^v^	2.9300
C8···O3^vi^	3.188 (4)	H8···H2	2.1900
C10···O1^vi^	3.347 (4)	H8···H6	2.4100
C11···N1^vi^	3.431 (5)	H8···O3^vi^	2.4300
C12···O3^xi^	3.418 (5)	H11···N2	2.6700
C13···C6^xii^	3.344 (6)	H11···H2	2.2300
C14···C1^xii^	3.576 (5)	H12···C14^xi^	3.0900
C5···H7C^ii^	3.0900	H12···O1^viii^	2.6100
C6···H7A	2.7800	H13···O2^vii^	2.6500
C6···H7B	2.7100	H14···O3	2.6400
			
C5—O2—C7	117.5 (3)	C10—C11—C12	118.9 (3)
C2—O1—H1	110 (3)	N3—C12—C11	124.1 (4)
N2—N1—C8	115.9 (3)	N3—C13—C14	124.3 (4)
N1—N2—C9	119.0 (3)	C10—C14—C13	118.4 (3)
C12—N3—C13	116.4 (4)	C2—C3—H3	120.00
C9—N2—H2	122 (3)	C4—C3—H3	120.00
N1—N2—H2	119 (3)	C3—C4—H4	119.00
C2—C1—C6	119.6 (3)	C5—C4—H4	119.00
C2—C1—C8	122.2 (3)	C1—C6—H6	120.00
C6—C1—C8	118.2 (3)	C5—C6—H6	120.00
O1—C2—C3	118.8 (3)	O2—C7—H7A	109.00
O1—C2—C1	122.4 (3)	O2—C7—H7B	109.00
C1—C2—C3	118.8 (3)	O2—C7—H7C	109.00
C2—C3—C4	120.7 (3)	H7A—C7—H7B	109.00
C3—C4—C5	121.3 (4)	H7A—C7—H7C	109.00
C4—C5—C6	119.0 (3)	H7B—C7—H7C	110.00
O2—C5—C4	115.9 (3)	N1—C8—H8	120.00
O2—C5—C6	125.1 (3)	C1—C8—H8	120.00
C1—C6—C5	120.6 (3)	C10—C11—H11	121.00
N1—C8—C1	120.9 (3)	C12—C11—H11	121.00
O3—C9—N2	123.0 (3)	N3—C12—H12	118.00
O3—C9—C10	121.9 (3)	C11—C12—H12	118.00
N2—C9—C10	115.1 (2)	N3—C13—H13	118.00
C11—C10—C14	117.9 (3)	C14—C13—H13	118.00
C9—C10—C11	122.9 (3)	C10—C14—H14	121.00
C9—C10—C14	119.1 (3)	C13—C14—H14	121.00
			
C7—O2—C5—C6	−4.1 (6)	O1—C2—C3—C4	179.3 (4)
C7—O2—C5—C4	175.8 (4)	C2—C3—C4—C5	0.5 (6)
N2—N1—C8—C1	−179.4 (3)	C3—C4—C5—O2	−179.3 (4)
C8—N1—N2—C9	−167.1 (3)	C3—C4—C5—C6	0.6 (6)
N1—N2—C9—C10	−176.4 (3)	O2—C5—C6—C1	179.5 (3)
N1—N2—C9—O3	1.7 (5)	C4—C5—C6—C1	−0.4 (5)
C13—N3—C12—C11	1.5 (6)	O3—C9—C10—C14	−30.5 (4)
C12—N3—C13—C14	−0.7 (6)	O3—C9—C10—C11	149.2 (3)
C8—C1—C6—C5	178.8 (3)	N2—C9—C10—C14	147.6 (3)
C6—C1—C2—C3	1.8 (5)	N2—C9—C10—C11	−32.7 (4)
C2—C1—C6—C5	−0.8 (5)	C14—C10—C11—C12	−1.8 (5)
C8—C1—C2—O1	1.3 (5)	C9—C10—C11—C12	178.5 (3)
C8—C1—C2—C3	−177.8 (3)	C9—C10—C14—C13	−177.8 (3)
C6—C1—C2—O1	−179.2 (3)	C11—C10—C14—C13	2.5 (5)
C2—C1—C8—N1	3.7 (5)	C10—C11—C12—N3	−0.3 (6)
C6—C1—C8—N1	−175.9 (3)	N3—C13—C14—C10	−1.4 (6)
C1—C2—C3—C4	−1.6 (6)		

Symmetry codes: (i) *x*−1/2, −*y*+3/2, *z*−1/2; (ii) *x*, −*y*+2, *z*−1/2; (iii) *x*−1, *y*, *z*; (iv) *x*−3/2, −*y*+3/2, *z*−1/2; (v) *x*−1/2, *y*+1/2, *z*; (vi) *x*+1/2, −*y*+3/2, *z*+1/2; (vii) *x*+1/2, *y*−1/2, *z*; (viii) *x*+3/2, −*y*+3/2, *z*+1/2; (ix) *x*−1/2, −*y*+3/2, *z*+1/2; (x) *x*, −*y*+2, *z*+1/2; (xi) *x*+1, *y*, *z*; (xii) *x*+1/2, −*y*+3/2, *z*−1/2.

### Hydrogen-bond geometry (Å, °)

**Table:**

*D*—H···*A*	*D*—H	H···*A*	*D*···*A*	*D*—H···*A*
O1—H1···N1	0.96 (4)	1.80 (4)	2.628 (4)	142 (4)
N2—H2···O3^vi^	0.88 (4)	2.03 (4)	2.872 (4)	161 (4)
C8—H8···O3^vi^	0.93	2.43	3.188 (4)	139

Symmetry codes: (vi) *x*+1/2, −*y*+3/2, *z*+1/2.
